# Molecular Evolution and Structural Features of IRAK Family Members

**DOI:** 10.1371/journal.pone.0049771

**Published:** 2012-11-14

**Authors:** Vijayakumar Gosu, Shaherin Basith, Prasannavenkatesh Durai, Sangdun Choi

**Affiliations:** Department of Molecular Science and Technology, Ajou University, Suwon, Korea; University of South Florida College of Medicine, United States of America

## Abstract

The interleukin-1 receptor-associated kinase (IRAK) family comprises critical signaling mediators of the TLR/IL-1R signaling pathways. IRAKs are Ser/Thr kinases. There are 4 members in the vertebrate genome (IRAK1, IRAK2, IRAKM, and IRAK4) and an IRAK homolog, Pelle, in insects. IRAK family members are highly conserved in vertebrates, but the evolutionary relationship between IRAKs in vertebrates and insects is not clear. To investigate the evolutionary history and functional divergence of IRAK members, we performed extensive bioinformatics analysis. The phylogenetic relationship between IRAK sequences suggests that gene duplication events occurred in the evolutionary lineage, leading to early vertebrates. A comparative phylogenetic analysis with insect homologs of IRAKs suggests that the Tube protein is a homolog of IRAK4, unlike the anticipated protein, Pelle. Furthermore, the analysis supports that an IRAK4-like kinase is an ancestral protein in the metazoan lineage of the IRAK family. Through functional analysis, several potentially diverged sites were identified in the common death domain and kinase domain. These sites have been constrained during evolution by strong purifying selection, suggesting their functional importance within IRAKs. In summary, our study highlighted the molecular evolution of the IRAK family, predicted the amino acids that contributed to functional divergence, and identified structural variations among the IRAK paralogs that may provide a starting point for further experimental investigations.

## Introduction

Interleukin-1 receptor-associated kinases (IRAKs) are intracellular kinases that belong to a family containing death domains (DDs). The IRAKs play important roles in signal transduction mediated by Toll-like receptors (TLRs) and interleukin-1 receptors (IL-1Rs) [1]. TLRs are crucial in the innate immune response to microbial pathogens because of their ability to recognize pathogen-associated molecular patterns (PAMPs) [2]. IL-1R and its family members, including IL-18R and IL-33R, are cytokine receptors that initiate and control inflammatory and immune responses. Unregulated TLR or IL-1R activation may lead to pathological conditions ranging from chronic inflammation to the onset of autoimmune disease. Several attempts have been made to modulate TLR/IL-1R responses, including direct blocking of receptor activation and inhibition of downstream signaling pathways [3], [4].

Upon stimulation, IL-1R and all TLRs, except TLR3 and certain TLR4 signals, recruit the Toll/IL-1 receptor (TIR) domain-containing adaptor molecule, myeloid differentiation factor 88 (MyD88), through a TIR-TIR homotypic interaction, leading to the activation of NF-κB [5]. In the case of TLR2 and TLR4, another TIR adaptor protein, Mal (also known as TIRAP), recruits MyD88 to the receptor complex [6], [7]. TLR3 can signal independently of MyD88 via the TRIF pathway, which induces the activation of interferon regulatory factors (IRFs) and the production of type I interferons [8], [9]. TLR4 can also signal through TRIF via a bridging adaptor protein called TRIF-related adaptor molecule (TRAM), resulting in a delayed MyD88-independent NF-κB response [10]. Formation of the TLR receptor-adaptor complex leads to the recruitment of IRAKs [11]. Upon recruitment, IRAK4 catalyzes the phosphorylation of key serine and threonine residues in IRAK1. This phosphorylation promotes the autophosphorylation of IRAK1, which then dissociates from the receptor complex and interacts with tumor necrosis factor receptor-associated factor 6 (TRAF6) and the TAK1-TAB1-TAB2 kinase complex, leading to the activation of NF-κB, p38, and JNK MAPKs [12], [13].

The term IRAK originally referred to a serine/threonine-specific kinase that could be co-precipitated in an IL-1 inducible manner with the IL-1 receptor [14], [15]. There are 3 ubiquitously expressed mammalian IRAK members: human IRAK1, IRAK2, and IRAK4. A fourth IRAK member, human IRAKM, is induced only in monocytes and macrophages [16], [17]. Both IRAK1 and IRAK4 are active Ser/Thr kinases, and phosphorylation of IRAK1 by IRAK4 is crucial for IRAK1 activation during TLR signaling [16], [18]. Mice lacking IRAK4 are completely resistant to lipopolysaccharide (LPS) treatment and display attenuated IL-1R, TLR2, TLR3, and TLR9-induced cytokine responses [19]. Conversely, although IRAK1 was originally thought to be crucial for TLR-induced NF-κB activation, IRAK1-deficient mice show only partial defects in IL-1, IL-18, and LPS-induced signaling [1], [20], [21], [22]. IRAK1 and IRAK4 are the only active kinases in the IRAK family because the Asp residue in the IRAK1/4 kinase domain (KD) is substituted with an Asn residue in IRAK2 and a Ser residue in IRAKM. However, like all other IRAK members, IRAK2 contains a functional ATP-binding pocket with a conserved Lys residue in the protein kinase subdomain [23]. This residue may be sufficient for IRAK2 to function as an active kinase [24]. An *in vitro* kinase assay showed that IRAK2 is phosphorylated upon stimulation with the TLR2 ligand [24]. Therefore, IRAK2 is no longer considered a pseudo kinase, and IRAKM is the only family member that lacks kinase activity. The most distinct feature of IRAKM is its function as a negative regulator of TLR signaling [12].

IRAK proteins share similar domain organization, with an N-terminal DD, a ProST domain, a central conserved KD, and a C-terminal domain (except for IRAK4, which lacks the C-terminal domain) [25], [26]. The DD is vital for signaling because it interacts with other signaling molecules, such as MyD88. However, IRAK members that lack the DD act as signaling antagonists [27], [28]. IRAK1 is hyperphosphorylated in the ProST region, which is rich in Ser, Pro, and Thr residues [26]. Additionally, the ProST domain contains 2 potential PEST sequences that may facilitate the degradation of IRAK1 [29]. The central KD contains an activation loop that is important for kinase activity. In recently solved crystal structures, the IRAK4 KDs exhibit structural features of both Ser/Thr and Tyr kinases [30], [31]. Furthermore, IRAK family members have a tyrosine gatekeeper residue at the center of the ATP-binding site. Lastly, the C-terminal end is important for the interaction with TRAF6 [32]. Interestingly, *Drosophila* has a single kinase homolog, Pelle, which is related to the mammalian IRAKs and is required for Toll signaling in arthropods [33]. Although the downstream signaling events differ between insects and humans, signaling in both organisms depends on inhibitor phosphorylation and proteolysis [34]. The Tube protein in *Drosophila* is another IRAK homolog that might have diverged from an IRAK ancestral gene; however, Tube does not contain a KD [35]. The domain organization of Tube comprises an N-terminal DD and a C-terminal 5-copy repeat of the Tube repeat (an 8 amino acid motif) [36]. The Tube DD acts as a bridge between the DDs of MyD88 and Pelle [37], [38]. The C-terminal Tube repeat mediates the stable association between the Dorsal and Tube proteins in embryos [39], [40].

Although IRAK members are conserved among species, they have undergone remarkable sequence, structural, and functional divergence. As more IRAK genes and crystal structures become available, molecular evolutionary analysis of IRAK members in a wide range of species is needed. In this paper, we describe a phylogenetic analysis to trace the evolutionary history of the IRAKs. Selection and functional divergence analyses were combined to interpret the relationship between the site-specific evolution and functional divergence of the IRAK family members. Furthermore, comparative modeling was utilized to build three-dimensional (3D) models of the IRAKs in order to investigate their structural variations.

## Methods

### Sequence data collection

The biochemically characterized IRAK protein sequences IRAK1 [16], IRAK2 [28], IRAKM [41], IRAK4 [18], Pelle, and Tube [36] from *Homo sapiens* and *Drosophila melanogaster* were retrieved from the National Center for Biotechnology Information (NCBI). To identify IRAK homologs, we utilized the above retrieved protein sequences as queries for BLASTP and TBLASTN [42]. The homologous search was performed by querying protein databases or genome assemblies at the NCBI (http://www.ncbi.nlm.nih.gov/sites/entrez), ENSEMBL (http://www.ensembl.org/Multi/blastview), SpBASE (http://www.spbase.org/SpBase/search), and UNIPROT (http://www.uniprot.org/blast) websites. For each IRAK, Pelle, and Tube protein, a BLASTP search with default parameters produced approximately 250 hits. We manually collected biochemically characterized IRAK proteins and used them as queries for repeated BLAST searches. From the BLAST output, we identified IRAK and Pelle (30% sequence identity), and Tube (20% sequence identity) homologs over a stretch of “X<Y<Z”. The X and Z values varied depending upon the specific IRAK protein, and Y corresponded to the length of the specific IRAK protein. The lengths of the IRAK proteins varied considerably; for example, IRAK1 is 712 amino acids (aa) long, whereas IRAK4 is 460 aa long. Because of the length discrepancies of the IRAK paralogs, we utilized a unique cut-off range for each IRAK homolog. The size ranges were as follows: IRAK1, 612–812 aa; IRAK2, 525–725 aa; IRAKM, 496–696 aa; IRAK4, 360–560 aa; Pelle, 401–601 aa; and Tube, 362–562 aa. Each newly identified putative or hypothetical sequence was used as a query for a BLAST search against the non-redundant GenBank database [43] to determine whether the best hit was an IRAK-, Pelle-, or Tube-encoding gene. After removing the redundant and partial sequences, the cut-off analysis produced 142 IRAK, 19 Pelle, and 19 Tube proteins. Interestingly, 7 kinases from arthropods (*Aedes aegypti, Danaus plexippus, Anopheles gambiae, Culex quinquefasciatus, Litopenaeus vannamei, Pediculus humanus corporis,* and *Daphnia pulex*) were also included in our analysis, and we named these kinases Tube-like kinases (TLKs). Six PIK-1 proteins were also included as Pelle homologs from nematodes because these proteins showed high sequence similarity to the Pelle protein. From the final dataset, we utilized the following taxonomic groups: Mammalia, Reptilia, Aves, Amphibia, Actinopterygii, Tunicata, Cephalochordata, Echinodermata, Insecta, Nematoda, Hemichordata, and Porifera. The final 193 sequences were subjected to InterProScan [44] and ScanProsite [45] analyses to determine the domain organization. Furthermore, all-against-all pairwise distances between the candidate sequences were calculated from a MAFFT alignment using the Geneious software (http://www.geneious.com).

### Prediction of potential binding and ubiquitination sites

MnM (Minimotif Miner, http://mnm.engr.uconn.edu/MNM/SMSSearchServlet) was used to search shared motifs and potential binding sites in the final dataset containing the IRAK protein sequences. CKSAAP_ubsite (http://protein.cau.edu.cn/cksaap_ubsite/) was used to identify the ubiquitination sites. PEST motifs were identified using epestfind from the EMBOSS package (http://emboss.bioinformatics.nl/cgi-bin/emboss/epestfind).

### Multiple sequence alignments and phylogenetic analysis

Multiple sequence alignment (MSA) is crucial for extracting evolutionary information from a large number of sequences. In our study, MSAs for coding sequences were performed using the Guidance web server [46]. The MSAs with all vertebrate IRAKs (142 sequences) and IRAK representatives with insect homologs (74 sequences) were conducted using MAFFT [47] and Clustal [48], as implemented in the Guidance web server (http://guidance.tau.ac.il/), using the default parameters. The reliability of the alignment was examined using the Guidance web server [46]. The ambiguous regions were removed using Jalview [49]. Of the 142 vertebrate IRAK sequences, 3 sequences were removed owing to their poor alignment. Therefore, the final alignment of vertebrate IRAKs contained 139 sequences. The MSAs were used to construct 2 phylogenetic trees using 2 independent approaches, neighbor joining (NJ) and Bayesian inference. The NJ tree was constructed using the Jukes-Cantor genetic distance model in the Geneious software, and the branch support was assessed with 1,000 bootstrap replicates. Bayesian inference was performed with the MrBayes v3.12 [50] plug-in implemented in the Geneious software. The MrBayes parameters were as follows: prset aamodelpr = mixed, lset rates = gamma, Ngammacat = 4, mcmcngen = 1,000,000, subsamplefre = 500, nchains = 4, starting tree = random. The branches were tested for significance by bootstrapping with 1000 replicates. Finally, the ConSurf server [51] was used to identify conserved IRAK residues.

### Selection analysis

Detection of selective forces acting on single amino acid sites in a given protein sequence may contribute to our understanding of the evolution of protein sequences. Therefore, we conducted site-wise Ka/Ks analysis of the IRAK sequences using the Selecton server v2.4 [52], [53], an online server-based program. The Selecton server uses a non-aligned file containing homologous coding DNA sequences or a codon-aligned file containing coding DNA sequences, in FASTA format, as input. We submitted aligned IRAK gene-coding sequences for each IRAK subfamily with the human sequence as a query. For the Pelle and Tube/TLK gene-coding sequences, the *D. melanogaster* sequence was used as a query.

### Functional divergence analysis

On the basis of the method suggested by Gu et al., the type I and II functional divergence of the IRAK gene sequence duplication events was tested using the Diverge 2.0 software [54], [55]. This method is based on a maximum likelihood procedure to estimate significant changes in the rate of evolution after the emergence of 2 paralogs. Type I divergence results in amino acid sites that are conserved in one group and diverse in another group. Sets of N-terminal DD and KD sequences from vertebrate IRAK proteins were individually analyzed. The test for type I divergence is based on the coefficient of divergence (*θ*). This coefficient corresponds to the probability that a specific site has undergone divergence in a pairwise comparison, i.e., *θ* = 0 indicates no functional divergence, increasing *θ* values indicate increasing functional divergence, and *θ* = 1 represents maximum divergence. The cut-off value for the posterior probability was determined by consecutively eliminating the highest scoring residues from the alignment until the coefficient of functional divergence reached 0. Type II functional divergence was evaluated for vertebrate IRAK KD sequences to identify residues with substantial biochemical changes in different IRAK subfamilies.

### Homology modeling

Comparative modeling was used to build 3D structures of the DDs and KDs of the IRAK proteins. To date, the IRAK DD crystal structures of IRAK2 (3MOP-K), IRAK4 (3MOP-G), Pelle (1D2Z-A), and Tube (1D2Z-B) have been solved. Templates were chosen for the target proteins based on sequence similarity. For the homology modeling of the IRAK1 DD, 3MOP-N was selected as a template because it shared a sequence identity of 31.1% with IRAK1. For homology modeling of IRAKM, 3MOP-G was chosen as a template because it shared a 33% sequence identity with IRAKM. For PIK-1 modeling, 1D2Z-A served as a suitable template because it shared a sequence identity of 35% with PIK-1. Based on the sequence identity (35% with IRAK1, 32% with IRAK2, 33% with Pelle, 35% with PIK-1, and 35% with TLK), 2NRY-A was chosen as a common template for the KDs of IRAK1, IRAK2, Pelle, PIK-1, and TLK 3D modeling. In contrast, for IRAKM KD modeling, 2NRU-A served as a suitable template with a sequence identity of 36%. The crystal structure of the IRAK4 KD is available (2NRY). Structure-based sequence alignment was performed using T-coffee expresso (http://tcoffee.crg.cat/apps/tcoffee/do:expresso), and this alignment was utilized for the target-template alignment. To generate 3D structural models of the DDs and KDs of the IRAK, Pelle, Tube/TLK, and PIK-1 proteins, we used a comparative modeling approach implemented in Modeller version 9v8 [56]. A series of 20 models were built, from which the final model was selected on the basis of stereochemical and energetic considerations. The final models were subjected to energy minimization by using the AMBER 03 force field for 2 picoseconds (ps), implemented in YASARA [57]. The stereochemical quality of these proteins was assessed using ProQ [58] and MetaMQAP [59]. In addition, molecular dynamics (MD) simulations were performed using GROMACS 4.5.5 software [60] for all the DD modeled structures. The individual structures were inserted in to a cubic box maintaining 10 Å between box edges and the protein surface. The system was then solvated with the simple point-charge water molecule and then minimized using the steepest descent method with a GROMOS96 43a1 force field. Counter ions (Na^+^ or Cl^−^) were added to neutralize the system. The temperature of the bath was set to 300 K, and the coupling time constant was set to 0.1 ps [61]. The box pressure was then maintained at 1 bar using a 1 ps time constant and a water compressibility of 4.5×10^−5^ bar^−1^. After equilibrating the system, a 10-nanoseconds (ns) production simulation was conducted with a 20-ps time step at a pressure of 1 bar and a temperature of 300 K.

## Results

### Identification of IRAK orthologs

There are 4 mammalian IRAK members but only 2 IRAK homologs in arthropods, Pelle [33] and Tube [35]. To perform a phylogenetic analysis of the entire IRAK gene family, we examined several genome databases by using biochemically characterized human IRAK sequences as queries (detailed in the Methods section). Orthologs of the IRAK genes are found in virtually all vertebrates for which genome sequences are available. During our database search for invertebrate IRAK orthologs, we identified a single IRAK gene, very similar to IRAK4, in representative species of some but not all invertebrate bilaterian lineages. A clear IRAK4 ortholog was identified in *Branchiostoma floridae* (Cephalochordata), *Ciona intestinalis* (Urochordata), *Strongylocentrotus purpuratus* (Echinodermata), *Saccoglossus kowalevskii* (Hemichordata), and *Amphimedon queenslandica* (Porifera). In *A. queenslandica*, we identified 2 IRAK4 proteins [62], one with 390 aa (XP_003388757.1) and the other with 475 aa (XP_003389195.1). The first sequence had high similarity (40% identity) with human IRAK4 and had only a KD, whereas the second sequence had both a DD and a KD and showed 39% sequence identity to human IRAK4. This indicates that a vertebrate-like IRAK protein with a DD exists in the poriferans. We included the first sequence in our analysis because it showed higher sequence homology. Similarly, we identified 2 IRAK4 gene products in *Strongylocentrotus purpuratus*, one with 680 aa and the other with 1035 aa. However, we included only the 680 aa (SPU_000073.1) protein in the analysis because this sequence was more similar to the human IRAK4 gene.

In addition, we used the Pelle and Tube sequences to collect ortholog sequences in arthropods. During this search, we identified a Pelle homolog, nematode PIK-1. In contrast, during the search for orthologs of Tube, we identified proteins containing KDs rather than Tube proteins in some of the arthropods; hence, we named these proteins Tube-like kinases (TLKs). A MAFFT alignment was performed with all collected protein sequences. A few redundant and incomplete sequences were removed, and the final dataset comprised 193 sequences, (Table S1) including 53 IRAK4, 30 IRAK1, 29 IRAK2, 30 IRAKM, 19 Pelle, 19 Tube, 7 TLK, and 6 PIK-1 sequences. All-against-all pairwise sequence alignments were conducted for all 193 sequences to determine the similarities across the entire IRAK family (Table S2). The MSA of the final data set was performed using the Guidance server (refer to the Methods section) [46].

### Phylogenetic analysis of vertebrate IRAK paralogs

**Figure 1:**
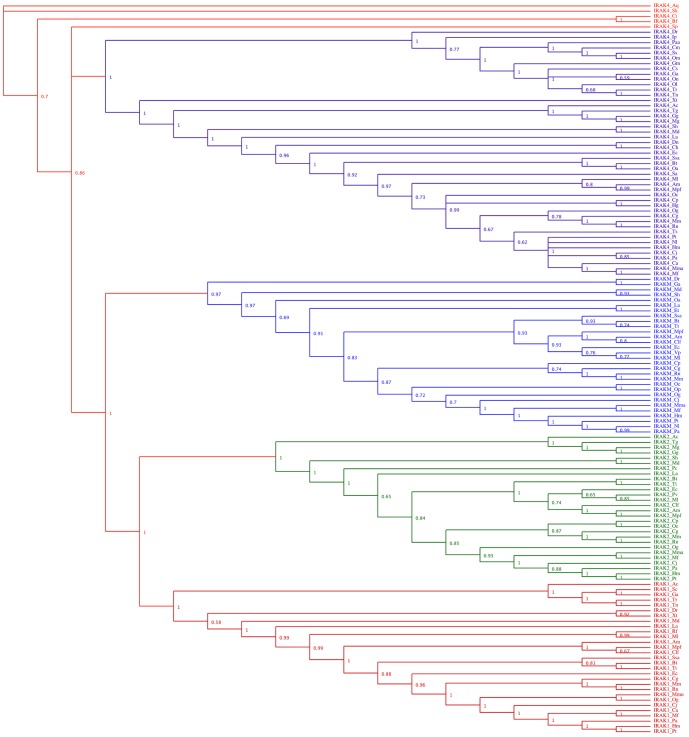
Phylogenetic relationship between vertebrate IRAK subfamilies determined using the Bayesian inference. A total of 139 IRAK coding sequences were included in this analysis. *A. queenslandica* was considered as an outgroup. The numerical values represent posterior probability. As depicted, the tree clustered into 4 major monophyletic clades. Each clade is represented by a unique color: IRAK4 (purple), IRAKM (blue), IRAK2 (green), and IRAK1 (light red). The species closest to the outgroup invertebrates are shown in red. Taxonomical convention: IRAK protein name followed by an abbreviated form of the species name.

To explore the phylogenetic relationship between the IRAK paralogs in vertebrates, we constructed a rooted phylogenetic tree using the MrBayes and NJ methods for 139 IRAK genes from approximately 100 species (Figure 1 and Figure S1). The phylogenetic analyses showed similar tree topologies and supported high posterior probability and bootstrap values for both MrBayes and NJ. Furthermore, the branches of the trees followed the accepted evolutionary order. Therefore, we selected both trees for further analysis. The results revealed an evolutionary partitioning of the IRAKs into 4 major clusters: IRAK1, IRAK2, IRAKM, and IRAK4. We utilized the *A. queenslandica* IRAK4 gene as an outgroup for the phylogenetic tree reconstruction because *A. queenslandica* is the most basal organism in the metazoan lineage [63]. In addition to *A. queenslandica*, we included 4 lower invertebrate species, *S. kowalevskii*, *S. purpuratus*, *C. intestinalis*, and *B. floridae*, in the phylogenetic tree reconstruction because all these invertebrate species are closely related to the vertebrate lineage. In the MrBayes phylogenetic tree, *A. queenslandica* and S. *kowalevskii* acted as an outgroup because *S. kowalevskii* belongs to the phylum Hemichordata and forms the lower duplicate of vertebrates in our data set. However, in the NJ tree, *A. queenslandica* rooted well with all the species in the tree. The Echinoderm *S. purpuratus*, Urochordate *C. intestinalis*, and Cephalochordate *B. floridae* represent the best pre-duplicative set of the vertebrate genome [64] and contain only 1 copy of the IRAK4 gene, which strongly suggests that gene duplication events have occurred in the lineage leading to the early vertebrates. All mammalian vertebrate species have 4 IRAK paralogs; however, a few non-mammalian vertebrates contain only 1 or 2 IRAKs. In the phylogenetic tree analysis, the evolution of IRAKs in vertebrates in each cluster recapitulated the phylogeny of the species. The phylogenetic analysis of vertebrate IRAKs suggested that the IRAK1 and IRAK2 diverged from the ancient IRAK4 by a gene-duplication event that occurred in early vertebrates. Another gene duplication event in the IRAK1/2 clade resulted in the divergence of the IRAK1 and IRAK2 subfamilies. The origin of the IRAKM subfamily can be rooted from one or more gene duplication events in the early mammalian lineage (Figure 1 and Figure S1).

Our current data set yielded interesting findings. All mammalian species in the data set have 1 gene that is orthologous to each of the IRAKs. In contrast, among the non-mammalian vertebrates, Aves lack IRAK1 and IRAKM, reptiles and amphibians lack IRAKM, and Actinopterygii lack IRAK2. However, the IRAKM is present in *Danio rerio* and *Gasterosteus aculeatus* (Actinopterygii) but not in other non-mammalian vertebrates. Furthermore, most of the 4 IRAKs duplicated and then diverged before the divergence of the earliest mammalian lineage. This is evident in both the platypus and eutherian genomes, which contain most of these gene duplicates, thereby suggesting that these genes diverged earlier than the species lineages. However, we excluded the platypus IRAK1 sequence from our analysis because it aligned poorly with the other IRAKs. Overall, our phylogenetic analysis indicated that both large-scale (genome-wide) and small-scale duplication events contributed to the evolution of the IRAK subfamilies. This conclusion is in agreement with previous findings that large-scale gene duplications occurred during chordate evolution [65],[66],[67].

### Phylogenetic analysis of IRAK paralogs and *D. melanogaster* homologs

**Figure 2:**
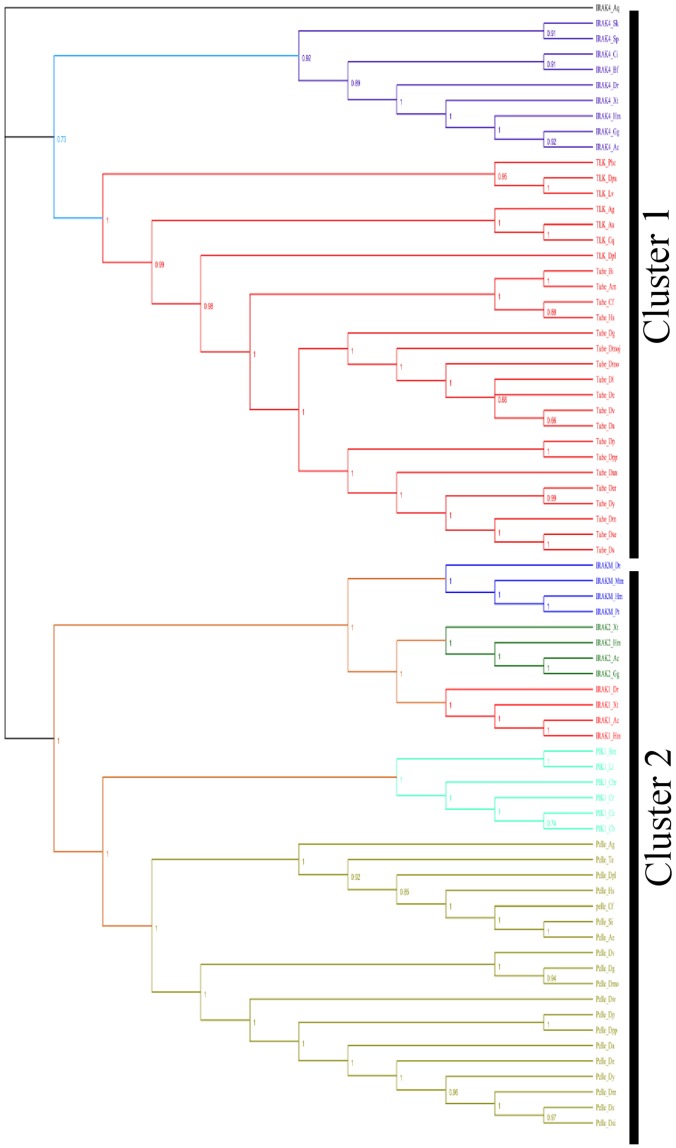
Phylogenetic analysis of IRAK genes compared with *D. melanogaster* homologs determined using the Bayesian inference. A total of 73 coding sequences were included in this analysis (22 representative vertebrate IRAK subfamily sequences based on taxonomy; 19 Pelle, 26 Tube/TLK, and 6 PIK-1 sequences). *A. queenslandica* was considered as an outgroup. The numerical values represent posterior probability. Each IRAK subfamily clade is represented by a unique color: Tube/TLK (hot pink), PIK-1 (cyan), and Pelle (khaki). Other IRAK colors are described in Figure 1. Taxonomic convention: IRAK protein name followed by an abbreviated form of the species name.

**Figure 3:**
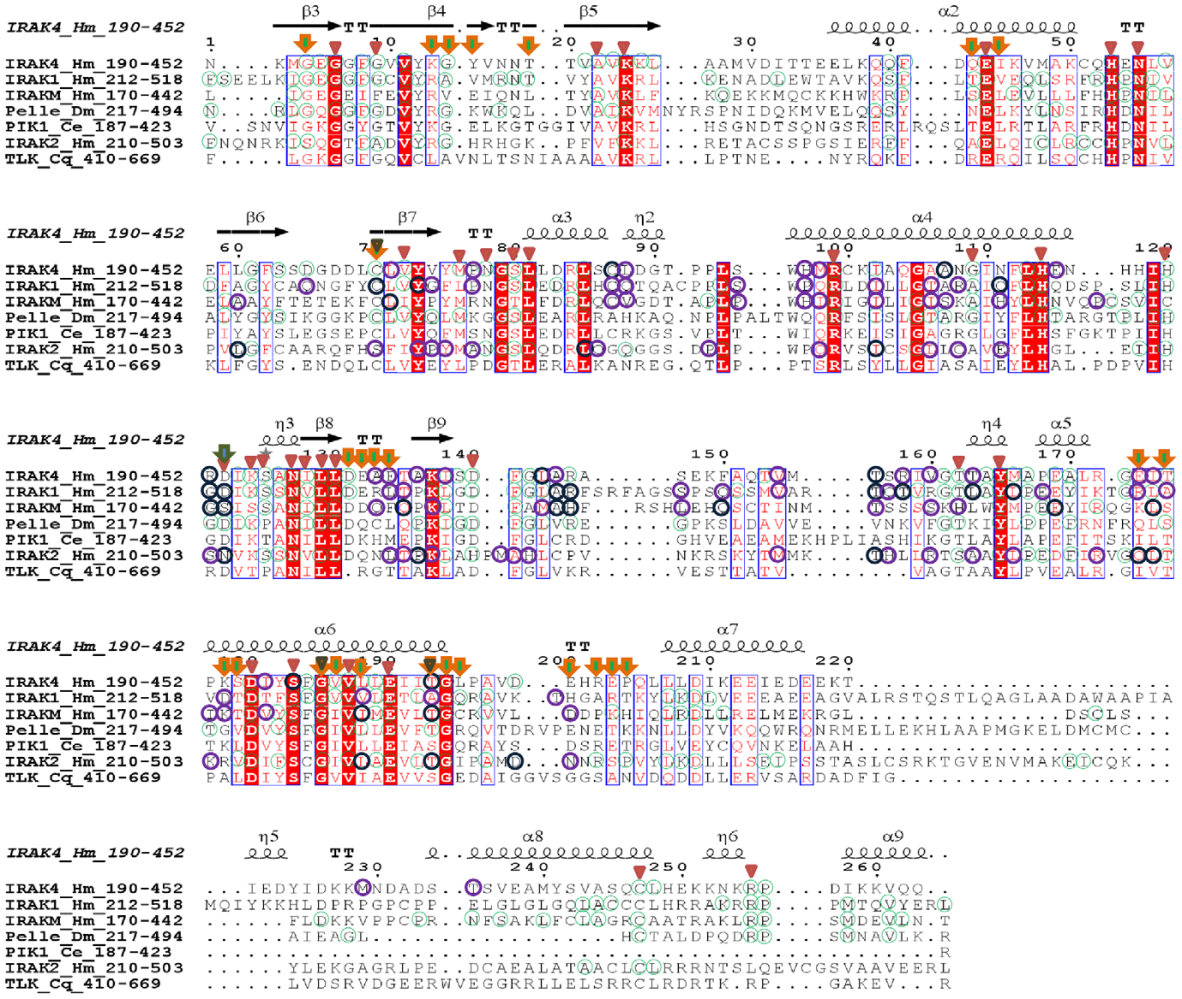
Multiple sequence alignment of the kinase domains (KDs) from representative IRAK family members along with the IRAK4 KD crystal structure. The amino acid numbers corresponding to the KD regions of each representative sequence are shown beside each IRAK protein name. Green colored circles represent the conservation observed within each subfamily in the species list. The potential type I sites are represented by purple circles, whereas conserved and type I potential sites are both depicted by dark-blue circles. The red triangles represent the conserved amino acids of all IRAK subfamilies from the entire species list. The green arrow represents the critical Asp residue. Orange arrows indicate potential type II sites. Gray triangles on the arrows represent conserved amino acids involved in type II divergence.

To explore the phylogenetic relationship between IRAK subfamily members and their homologs in arthropods and nematodes, we constructed a rooted phylogenetic tree using MrBayes and NJ methods with representatives of the IRAK subfamily, Pelle orthologs, Tube/TLK orthologs, and nematode orthologs (74 sequences). We utilized the *A. queenslandica* IRAK4 gene as an outgroup. The phylogenetic analysis indicated 2 major clusters of vertebrates, arthropod, and nematode homologs, including IRAK4, Tube/TLK, IRAKM, IRAK2, IRAK1, PIK-1, and Pelle (Figure 2 and Figure S2). In both Bayesian and NJ inferences, the IRAK4 gene from *A. queenslandica* clustered with all other family members, suggesting that the IRAK4 gene is an early offshoot of the IRAK members. In the phylogenetic tree, cluster 1 contained 2 groups, IRAK4 and Tube/TLK (Figure 2 and Figure S2). Generally, protein kinases can be classified as RD or non-RD based on a single position in the sequence of subdomain VI [68], [69], [70]. In RD kinases, an Arg residue precedes the invariant Asp residue that is vital for catalytic activity. Upon phosphorylation of the kinase activation loop region, the interaction between the Arg residue and the phosphate group converts the RD kinase from an inactive to an active state. On the other hand, the catalytic activity of the non-RD kinase does not require phosphorylation in the activation loop region. Moreover, the MSA of the representative KDs showed that both TLK and IRAK4 have an Arg residue before the Asp residue and hence are RD kinases (Figure 3). By contrast, in all the other IRAK members, Arg is substituted with Gly, except in IRAK2, in which Arg is substituted with Ser. Hence, the other IRAK members are non-RD kinases (Figure 3). Therefore, from the phylogenetic tree and MSA, Tube/TLK may represent a direct arthropod homolog of IRAK4. These results are consistent with previous reports demonstrating that Tube is an IRAK4 homolog [35]. In cluster 2, the Pelle protein from arthropods and the PIK-1 protein from nematodes are significantly more similar to IRAK1, IRAK2, and IRAKM in vertebrates than to any other invertebrate homologs (posterior probability of 1 and bootstrap value of 90.6%), thereby suggesting that Pelle and PIK-1 are homologs of either IRAK1, IRAK2, or IRAKM. The MSA of the KDs shows that Pelle, PIK-1, IRAK1, IRAK2, and IRAKM belong to the non-RD kinases. However, in IRAK2 and IRAKM, the catalytic Asp residue is substituted with Asn and Ser, respectively (Figure 3). Thus, Pelle and PIK-1 may be homologs of IRAK1 rather than IRAK2/M. In cluster 2, PIK-1 from nematodes and Pelle from arthropods are significantly related (posterior probability of 1 and bootstrap value of 100%). As a result, arthropods and nematodes are integrated into one group, assuming that the Ecdysozoa hypothesis is true [71], [72], [73]. The overall tree topology is well supported by a high posterior probability ranging from 0.73 to 1 and bootstrap values ranging from 66% to 100%. Additionally, the branching order of the IRAK subfamily fits well with the evolution of the species. It is clear from our analysis that Pelle and Tube/TLK proteins are present only in arthropods, and PIK-1 is present only in the nematode lineage (Table S1). IRAK4 is present in both vertebrates and invertebrates; remarkably, *A. queenslandica* and *S. kowalevskii* appear to have an IRAK4 gene. The sporadic appearance of the IRAK4 gene during evolution may be due to its distinct properties among the IRAK subfamily. Thus, our phylogenetic analysis performed using different methods indicates that the IRAK subfamily might have diverged and duplicated from a unique ancestral IRAK4-like kinase in the metazoan lineage. Furthermore, our results suggest that gene duplication events preceded the divergence of invertebrates and vertebrates.

### Comparative analysis of IRAK sequences

To interact with TRAF6, the C-terminal domain of IRAK1, IRAK2, and IRAKM (but not IRAK4, which lacks the C-terminal domain) is important [32]. IRAK1 contains 3 TRAF6 interaction motifs, IRAK2 has 2, and IRAKM has 1 [32]. Using the MnM server (http://mnm.engr.uconn.edu/MNM/SMSSearchServlet), we searched for TRAF6 binding motifs in all vertebrate IRAK members except IRAK4. MnM analysis showed that all vertebrate IRAK subfamily members, except IRAK4, have TRAF6-binding sites. IRAK1 has 3 potential TRAF6-binding sites, whereas some of the non-mammalian IRAK1 vertebrate species have 1–2 TRAF6-binding sites that are differentially distributed within the C-terminal domain of IRAK1. IRAK2 has 2 TRAF6-binding sites in all mammalian species. Similarly, IRAKM has 2 TRAF6-binding sites in most mammalian species, in both the C-terminal and ProST domains (Table S3). These potential TRAF6-binding sites on the IRAK1, IRAK2, and IRAKM proteins were conserved in all species. Furthermore, we analyzed the TRAF2-binding sites of all vertebrate IRAK family members (Table S3) using the MnM server.

We identified ubiquitination sites by searching the CKSAAP server for all IRAKs. The searches identified possible ubiquitination sites with high confidence scores and revealed that all IRAK proteins contain ubiquitination sites in the C-terminal domain. However, we found some ubiquitination sites in the ProST domain between the N-terminal DD and the central KD. In addition, the PEST motif is common among proteins that turnover rapidly in cells. The ProST domain of IRAK1 contains 2 potential PEST motifs that may facilitate its degradation [29]. In our searches, we identified PEST motifs in the C-terminal domains of IRAK1 and IRAK2 in all species (Table S3). No functional studies of C-terminal PEST motifs are currently available; hence, future biochemical studies are required to clarify their function. In a few species, IRAKM contained no PEST motifs. In IRAK4, the PEST motifs were located in the ProST domain between the N-terminal DD and the central KD (Table S3).

### Selective pressure on amino acid sites

Selection forces acting on individual codons within the vertebrate IRAK sequences were identified using an online web server, Selecton v2.4 [52], [53]. We estimated the number of synonymous (Ks) and non-synonymous (Ka) nucleotide substitutions per site for all vertebrate IRAK genes. To test for the presence of positive selection at the individual codon level for each IRAK subfamily member in vertebrates, likelihood rate tests were performed for models M8 (positive selection-enabled evolutionary model, beta+ω≥1) and M8a (null model with no positive selection, beta+ω = 1). The M8 evolutionary model showed that the overall Ka/Ks ratio was <1 for all IRAK subfamily members except for IRAK4. This indicates that the IRAK proteins have been subjected to strong purifying selection pressure (data not shown). Moreover, the M8a null model showed statistical significance for the IRAK4 subfamily. The major sites subjected to positive selection in IRAK4 were Gln 124, Met 125, Cys 128, Asp 131, Leu 134, Val 136, Tyr 146, Met 147, and Lys 156. Interestingly, the sites that had been subjected to positive selection in IRAK4 were located in the ProST domain of the IRAK4 gene. However, no positive selection of amino acids was identified in the DD or central KD regions. This shows the functional importance of the DD and KD. Most of the residues in these regions are highly conserved; hence, they act as a strong driving force in the evolution of the IRAK subfamilies.

### Functional divergence in the IRAK gene family

Gene duplication events provide a means to develop novel biological functions, and the changes in protein function may cause different functional constraints on the subsequent evolution of the duplicated genes. Generally, the functional divergence of a protein family occurs after major evolutionary events, such as speciation or gene duplication [74]. A type I functional divergence occurs shortly after gene duplication because of site-specific changes in the evolutionary rate between paralogous clusters. In contrast, type II functional divergence occurs later after gene duplication, when the evolutionary rate is constant [54], [75]. To elucidate the relationship between gene evolution and functional divergence, type I and type II divergences were examined. In this functional analysis, we considered only the N-terminal DD and central KD of the IRAK proteins. To reduce the computational complexity of the MSA, we aligned the DD and KD sequences separately. The coefficient of evolutionary functional divergence (*θ*), standard error, and maximum likelihood ratio (LRT) were determined for each pairwise comparison (Tables 1 and 2). The type I functional divergence showed medium/high *θ_I_* values for both the DD and KD for all pairwise comparisons. However, with a few exceptions, the other comparisons showed a *θ*>0 (*p*<0.05), suggesting that a site-specific rate shift is common after gene duplication.

**Table 1:** Type I evolutionary functional divergence between vertebrate IRAK subfamilies (death domain).

IRAK Proteins (DD)	IRAK1	IRAK2	IRAKM	IRAK4
**IRAK1**		0.7896±0.2450	0.0824±0.8465	0.8168±0.2072
**IRAK2**	**10.386337**		0.2375±0.9680	0.7288±0.2610
**IRAKM**	0.009475	ND		0.5072±0.7827
**IRAK4**	**15.5321**	**7.7949**	0.4198	

Type I functional divergence coefficients (*θ_I_*) and their respective standard errors are shown above the diagonal (upper right). Functional divergence values are represented by 1>*θ_I_*>0.5. Below the diagonal (lower left) are the likelihood ratio (LRT) values to test the null hypothesis of *θ_I_* = 0. The LRT values in bold significantly rejected the null hypothesis (p≤0.05).

**Table 2:** Type I evolutionary functional divergence between vertebrate IRAK subfamilies (kinase domain).

IRAK Proteins (KD)	IRAK1	IRAK2	IRAKM	IRAK4
**IRAK1**		0.5136±0.072459	0.6528±0.0681	0.4272±0.0676
**IRAK2**	**50.2416**		0.5±0.069232	0.564±0.1027
**IRAKM**	**91.7940**	**52.1583**		0.3961±0.0941
**IRAK4**	**39.8428**	**30.1119**	**17.7049**	

Type I functional divergence coefficients (*θ_I_*) and their respective standard errors are shown above the diagonal (upper right). Functional divergence values are represented by 1>*θ_I_*>0.5. Below the diagonal (lower left) are the likelihood ratio (LRT) values to test the null hypothesis of *θ_I_* = 0. LRT values in bold significantly rejected the null hypothesis (p≤0.05).

**Figure 4:**
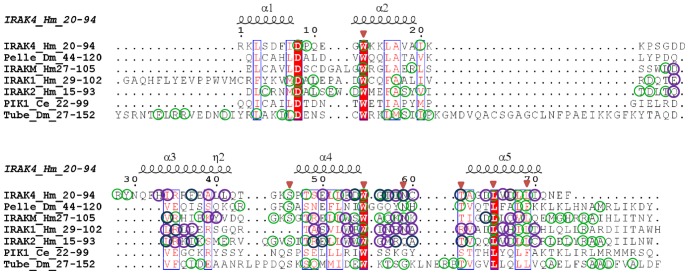
Multiple sequence alignment of the death domains (DDs) of representative IRAK family members along with the IRAK4 DD crystal structure. The amino acid numbers corresponding to DD regions for each representative sequence are shown beside the IRAK protein names. Green colored circles represent conservation within each IRAK subfamily. The potential type I sites are represented by purple circles, whereas conserved and type I potential sites are both depicted as dark blue circles. The red triangles represent conserved amino acids in all IRAK subfamilies from the entire species list.

**Figure 5:**
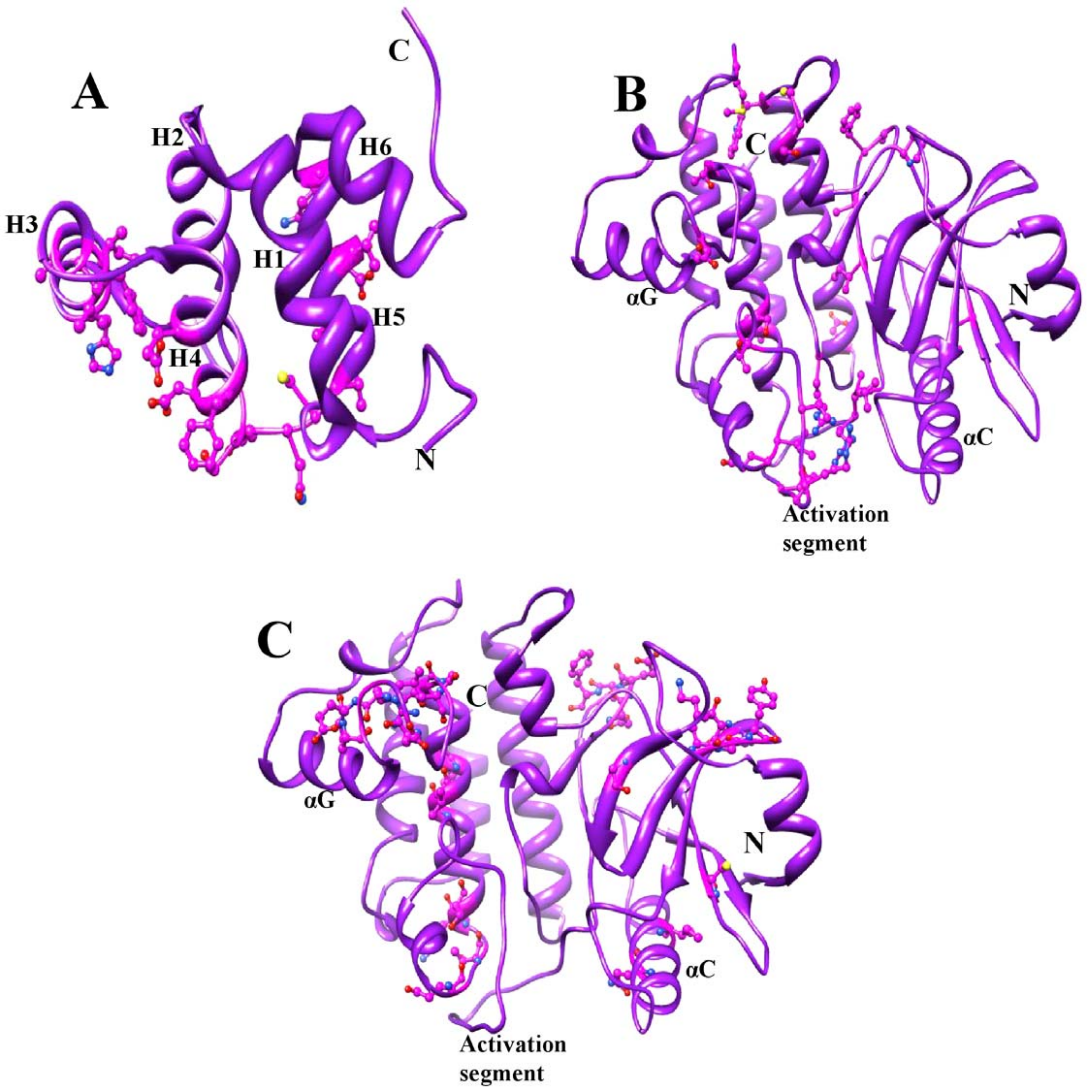
IRAK4 DD and KD structures. (A) The crystal structure of the IRAK4 DD and the Type I potential sites are shown using a ball-and-stick representation as a representative for all the DDs. (B) The crystal structure of the IRAK4 KD and the Type I potential sites are shown using a ball-and-stick representation as a representative for all the KDs. (C) Type II potential sites are shown using a ball-and-stick representation with reference to the IRAK4 KD crystal structure.

Further analysis focused on the pairwise functional differences between IRAK family members: IRAK4/IRAK1, IRAK4/IRAK2, IRAK4/IRAKM, IRAK1/IRAK2, IRAK1/IRAKM, and IRAK2/IRAKM. To identify the amino acids responsible for the functional divergence of the IRAK family members, we compared significant values of *θ_I_* using a posterior probability analysis with suitable cut-off values. Because of the variations in the *θ_I_* values between different pairs of clusters, we assigned a different cutoff value to each pairwise comparison. We identified several potential type I amino acid sites for all pairwise comparisons (Table S4). Most of the predicted type I sites clustered densely to helix 3 and helix 4 of the DD and were evenly distributed in the activation loop and helix αG of the KD (Figures 3, 4, 5A, and B). Generally, DD-DD interactions are crucial for the initiation of downstream TLR signaling events. Interestingly, the predicted potential type I sites Glu 69, Thr 76, and Asn 78 of the IRAK4 DD interact with the MyD88 DD [76]. Likewise, IRAK2 DD residues Met 66 and Arg 67 are important for the interaction with the IRAK4 DD, facilitating the formation of the myddosome complex [76]. Amino acid residues that correlated with substantial biochemical changes were identified by type II functional divergence analysis. We performed pairwise comparisons of the vertebrate IRAK KDs by using Diverge 2.0. In contrast to the type I functional divergence, evidence for type II functional divergence was not observed (data not shown) for any pairwise comparison (small *θ_II_* values). Notably, most of the residues received very low scores, indicating that only a few amino acids were associated with biochemical changes. Although there was no clear support for type II functional divergence (p>0.05), we performed an additional analysis to determine whether any potential site showed evidence for type II functional divergence. We assumed that a *posteriori* ratio test value (of an amino acid site) of >1.5 is a potential type II site. According to this assumption, 26 conserved amino acid sites showed typical shifts in amino acid properties (Figure 5C and Table 3). Most of the type II residues were evenly distributed in the conserved structural elements of the KD, such as β-strands of the N-lobe, helix αC, loops adjoining the helix αG, and in the activation segment. Overall, the type I and type II functional divergence analyses (Tables 1, 2, and 3) suggest that the IRAK genes are functionally divergent because of the different evolutionary rates and amino acid properties at specific sites. Hence, the functional divergence of these proteins may reflect long-term selective pressure.

**Table 3:** Overview of the kinase domain amino acid changes in 26 predicted sites during type II functional divergence.

Residue position (Human IRAK4)	Amino acid change	Property change
**193**	G→S (IRAK2)	Hydrophilic/hydrophilic
**202**	K→R	+/+
**203**	G→V	Hydrophilic/hydrophobic
**204**	Y→E	Hydrophilic/−
**209**	T→T,V (IRAK1)	Hydrophilic/hydrophilic
**232**	Q→S,T (IRAK1), A (IRAK2)	Hydrophilic/hydrophilic
**234**	I→L,V (IRAK1)	Hydrophobic/hydrophobic
**259**	C→C,S (IRAK2)	Hydrophilic/hydrophilic
**320**	D→D	−/−
**321**	E→D	−/−
**322**	A→Q	Hydrophobic/hydrophilic
**323**	F→F,L (IRAK1/2)	Hydrophobic/hydrophobic
**363**	E→K,Q (IRAK2), R (IRAK1)	−/+
**365**	T→S,A (IRAK1)	Hydrophilic/hydrophilic
**367**	K→K,D (IRAK1)	+/+
**368**	S→T,V (IRAK2)	Hydrophilic/hydrophilic
**374**	G→G	Hydrophilic/hydrophilic
**375**	V→I	Hydrophobic/hydrophobic
**377**	L→I	Hydrophobic/hydrophobic
**382**	T→T,A (IRAK1)	Hydrophilic/hydrophilic
**383**	G→G	Hydrophilic/hydrophilic
**384**	L→C,Q (IRAK1)	Hydrophobic/hydrophilic
**389**	E→D	−/−
**391**	R→P	+/Hydrophobic
**392**	E→K	−/+
**393**	P→H	Hydrophilic/+

Sites that correlated with substantial biochemical change were detected in the pairwise type II functional analysis of the vertebrate IRAKs (KD). The residue numbering is based on the human IRAK4 KD sequence. The amino acid changes between the subfamilies along with their property changes are provided. (“+” represents a positive amino acid whereas “−” represents a negative amino acid).

### Superimposition of IRAK DDs and KDs

In addition to the molecular evolution of IRAKs, we were interested in the structural similarity among IRAK proteins. Structurally, the IRAK family members share a similar domain organization. The DD and KD regions are functionally important. In order to understand the structural similarities and variations within the IRAK family, we used homology modeling to build 3D structures of the DDs and KDs for the IRAK members, except for IRAK2, IRAK4, the Pelle and Tube DDs, and the IRAK4 KD for which the crystal structures are available (refer to the Methods section). The geometry, configuration, and energy distribution were evaluated for the final models (Table S5). The DD structure is comprised of a 6-helix bundle fold (H1–H6) (Figure S3). Most of the potentially diverged amino acids identified from the functional divergence analysis of the IRAK DD were located in helix 3 and helix 4. These residues are not conserved among the IRAK DDs (Figure 4). Particularly, helix 3 and helix 4 are important for DD-DD interactions in initiating downstream TLR signaling. In addition, we subjected the DD models to MD simulations in order to assess their stability. Figure S4 depicts the backbone RMSD plot for the Cα-atoms with reference to the initial structure and as a function of time. The plot shows that all the DDs reached an equilibrium state after 3.5 ns.

**Figure 6:**
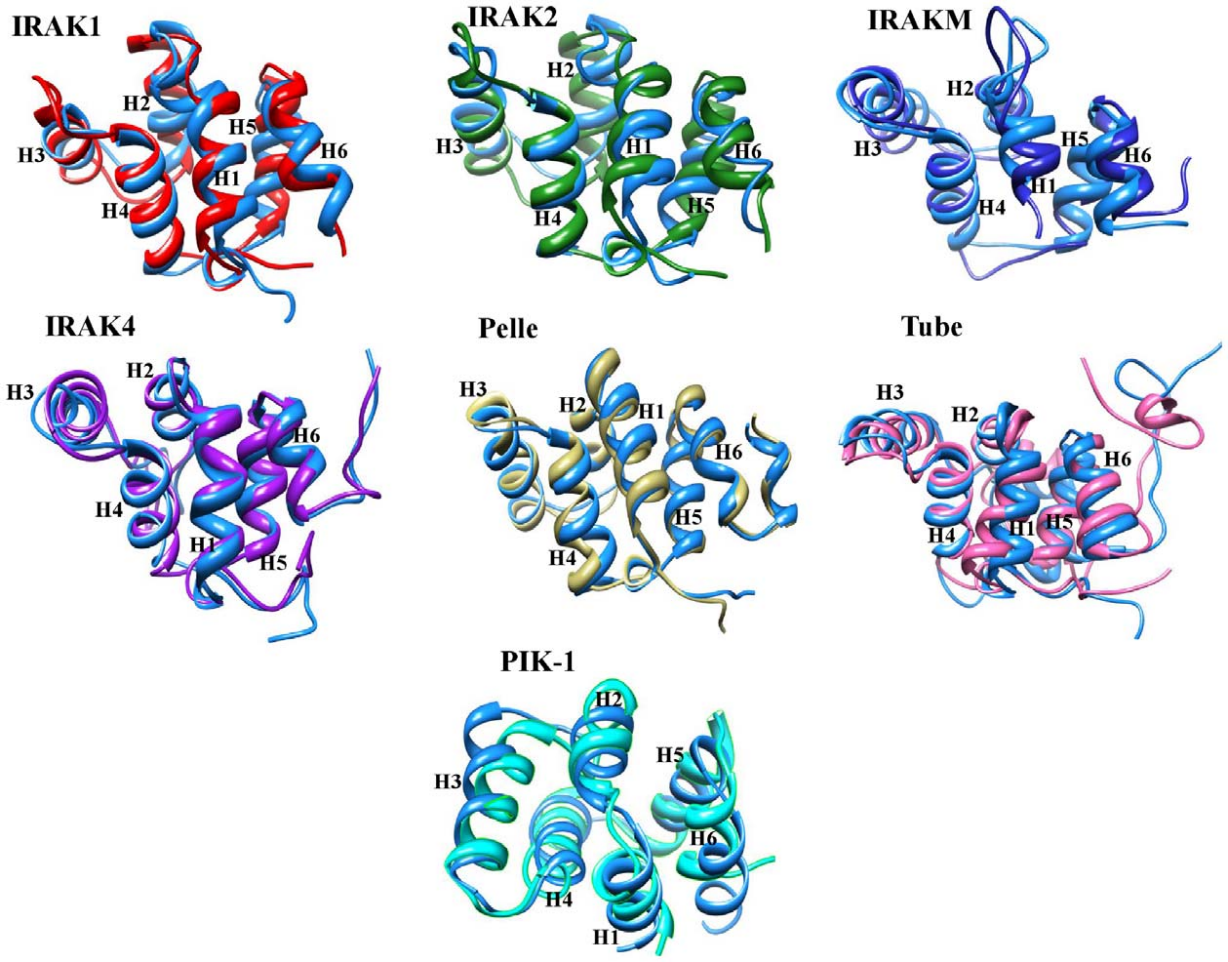
Superimposition of the IRAK DD final structures obtained from MD simulations with initial structures. Differences in the final structures of IRAK1, IRAK2, IRAKM, IRAK4, Pelle, Tube, and PIK-1 are shown. Their respective initial structures are in golden blue. The major structural variations are observed from helix 2 to helix 4.

**Figure 7:**
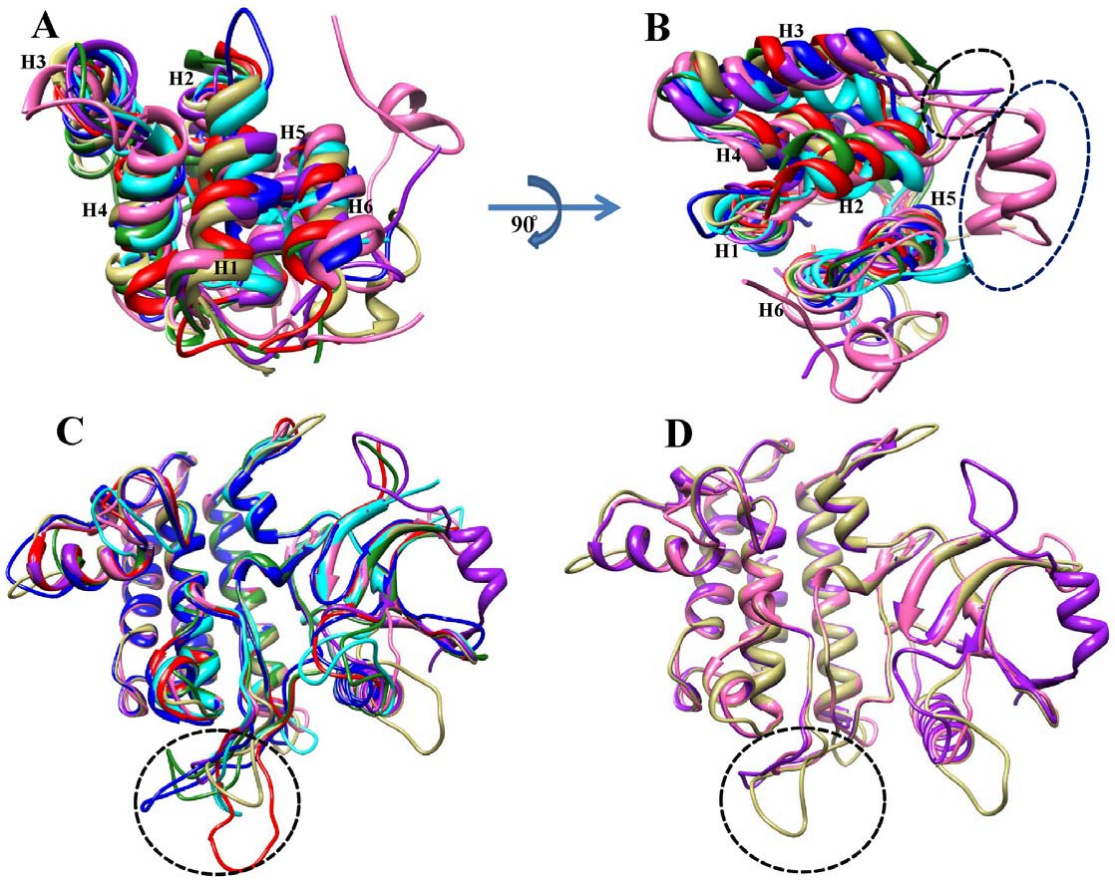
Superimposition and structural variation in the DD and KDs of IRAK subfamily members. (A) Structural superimposition of DDs. (B) Black- and blue-dotted circles representing the compact Ω loop containing 2 short helices (HA and HB) and a long loop between helix 2 and helix 3 in the IRAK4 DD. The IRAK protein colors are described in Figures 1 and 2. (C) Superimposition of all the kinase domains (KDs) of IRAK subfamily members. (D) Structural superimposition of IRAK4, Pelle, and TLK KDs. The structural alignment of the activation segment is shown using a black-dotted circle.

Superimposition of the final refined structure on the initial structure for all the DDs depicted in Figure 6 shows the following structural variations: 2.33 Å within helix 2–4 for IRAK1, 1.41 Å within helix 2–4 for IRAK2, 1.51 Å within helix 3–4 for IRAKM, 1.51 Å within helix 2–4 for IRAK4, and 0.92 Å, 2.24 Å, and 2.18 Å within helix 2–4 for Pelle, PIK-1, and Tube, respectively. The major structural variations were observed in helix 3 and helix 4 and in the loop regions between helices 2, 3, and 4. In addition, the superimposition of all the refined DD structures shows an RMSD of 2.34 Å based on 70 atoms (Figure 7A and B). Thus, we propose that although the DDs are structurally similar, a few differences in the loop region and a major difference in the orientation of the helices are critical for the binding specificity of each IRAK DD [76]. The compact loop region in the Tube protein with 2 short helices is not present in the DD family, and this insertion is poorly conserved among the Tube orthologs [37]. This insertion may block the intermolecular interaction surface within the Pelle and Tube dimer [37].

The modeled KD structures are depicted in Figure S5. The identified potentially diverged amino acids are evenly distributed in all the conserved elements. The modeled KD structures adopted a classical protein kinase fold, with an N-terminal lobe consisting of a 5-stranded, antiparallel β-sheet and a helix, as well as an α-helical C-terminal lobe (Figure S5) [30]. Figure 3 shows that IRAK1, IRAK2, and Pelle contain an insertion of approximately 20 aa in the helix αG adjoining-loop, whereas IRAK4 and TLK have a deletion in that region. Structural superimposition of the IRAK, Pelle, PIK-1, and TLK KDs (RMSD of 0.386 Å based on 206 atoms) showed that all KDs are structurally similar (Figure 7C). The RMSD between Pelle and IRAK4 was 0.364 Å, whereas the RMSD between TLK and IRAK4 was 0.386 Å. This observation suggests that the IRAK4 KD is structurally similar to the TLK KD as well as to the Pelle KD (Figure 7D). However, the activation loop of Pelle shows few insertions in the sequence; therefore, it did not align well with the IRAK4 KD. The structural superimposition of the IRAK1, IRAK2, IRAKM, and IRAK4 KDs showed an RMSD of 0.203 Å for 216 atoms, suggesting a high structural similarity among all IRAKs, although IRAKM has no kinase activity [12].

## Discussion

IRAKs are important in TIR-mediated signal transduction. Although IRAK proteins are structurally similar, several biochemical studies have shown that each IRAK member plays a non-redundant role in TLR/IL-1R signaling [12], [16], [19], [28]. Therefore, the IRAK proteins may be considered as potential therapeutic targets for the treatment of immune-related diseases. There are 4 IRAK proteins in mammals and an IRAK homolog, Pelle, in *Drosophila* [33]. Tube is also considered a homolog of the mammalian IRAKs [35]. However, Tube has no KD. We performed phylogenetic analysis to obtain insights into the evolutionary history of IRAK proteins and to dispel the long-standing controversy regarding the *Drosophila* homolog relationship with the mammalian IRAK members.

In this study, we assessed IRAK, Pelle, and Tube homologs from vertebrates and insects, and we found that most vertebrates have considerably more IRAK genes than invertebrates (Table S1). The vertebrate IRAKs have likely been subjected to multiple duplication events, at least before the teleosts (Figure 1 and Figure S1). Interestingly, after extensive gene duplication events, not all vertebrates contain 4 IRAK genes, likely due to the following 2 reasons. First, not all vertebrate genome sequences were included in our analysis. Second, some vertebrate species may have experienced gene loss, as observed in Aves species that lack IRAK1 and IRAKM, reptiles and amphibians that lack IRAKM, and teleosts that lack IRAK2 (Figure 1 and Figure S1) (Table S1). Surprisingly, we found IRAKM in *D. rerio* and *G. aculeatus*, although this gene is absent in other non-mammalian vertebrates. In addition, rodent species containing the IRAKM gene showed an Asn residue at the catalytic site, whereas non-rodent species had a Ser residue at this position. Hence, this sequence analysis indicates that IRAKM may act as a kinase like IRAK2 in rodent species, as IRAK2 has an Asn residue at the catalytic site. Therefore, further investigations are necessary to clarify the evolution of the IRAKM gene. These results suggest that a burst of gene duplication events and subsequent widespread gene deletion events occurred during the early evolution of vertebrate IRAKs. We identified 2 IRAK4 genes in *A. queenslandica* [62]; this potential ancestral IRAK4 gene may have evolved in primitive metazoans. In addition, some of the lower invertebrates, before the vertebrate lineage, have a single IRAK4 gene (Figure 1 and Figure S1). Four clusters were identified by vertebrate phylogenetic analysis, including IRAK1, IRAK2, IRAKM, and IRAK4 (Figure 1).

To understand the evolutionary origin of the vertebrate IRAK family, we included some lower invertebrates and the *A. queenslandica* sequence as an outgroup. Our results suggest that the current vertebrate IRAK genes share the IRAK4-like gene as a common ancestor in the metazoan lineage and that IRAK1 and IRAK2 duplicated earlier than IRAKM. The conservation of the 4 IRAK genes in mammals suggests that all 4 IRAK proteins are important in immune processes. Moreover, IRAKM is present only in mammalian species, suggesting that IRAKM has a specific role as a negative regulator of TLR signaling [12]. In addition, IRAKM is only detectable in monocytes and macrophages [16], [17]. Overall, the multiplicity of the IRAK proteins may have arisen partly by 2 rounds of genome duplication [65], [66], [67]. Furthermore, comparative phylogenetic analysis of vertebrate IRAKs with insect and nematode homologs showed 2 separate clades, including IRAK4, Tube/TLK, IRAKM, IRAK2, IRAK1, PIK-1, and Pelle (Figure 2 and Figure S2). The subclades IRAK4 and Tube/TLK are closest to each other and share a common ancestor in the IRAK4-kinase-like gene, suggesting that Tube/TLK is the closest homolog of IRAK4 and that Pelle is not a homolog of IRAK4 [35]. TLK and IRAK4 are RD kinases; moreover, the pairwise sequence identity of the IRAK4 and TLK KDs is higher than that of the Pelle and IRAK4 KDs (Table S6). This result is consistent with earlier reports suggesting that Tube is an IRAK4 homolog [35]. Other subclades, including IRAKM, IRAK2, IRAK1, Pelle, and PIK-1, form a major cluster and share a common ancestor in the IRAK4-kinase-like gene. Within the cluster, the Pelle and PIK-1 subclades are the closest, consistent with the Ecdysozoa hypothesis [71], [72], [73]. Because IRAKM, IRAK2, and IRAK1 form another subcluster within the cluster, Pelle/PIK-1 may be a homolog of IRAK1, IRAK2, and IRAKM. Of these proteins, Pelle, PIK1, and IRAK1 are non-RD-containing kinases, suggesting that Pelle is a homolog of IRAK1. In summary, the presence of the IRAK4 in Porifera suggests that this kinase is an ancestor in the metazoan lineage for the IRAK family. In addition, from our analysis, we propose that Tube/TLK is a homolog of IRAK4 and that Pelle is a homolog of IRAK1.

After gene duplication, processes such as pseudogene formation [77], sub-functionalization [78], and neo-functionalization [79] may result in altered functional constraints on gene family members. To identify the functional divergence of ancestral IRAKs, we performed rigorous type I and II functional divergence analyses. These analyses showed that type I rather than type II functional divergence is the main mechanism of divergence (Tables 1, 2, and 3). In this study, the divergence of amino acid residues of different IRAK subfamilies suggests that the IRAK proteins have diverse physiological functions. To further characterize the relationship between the site-specific evolution of amino acids and functional divergence, the possible amino acid site-related type I and II functional divergence was selected and mapped onto the protein models, the reference structure, and the sequence alignment. Posterior probability analysis of the pairwise comparison of IRAK paralogs identified significant functional divergence of the DDs and KDs. The amino acid residues identified in the type I functional divergence analysis, which are responsible for the strong purifying selection of the IRAK members, are predominantly located in helix 3 and helix 4 (Figure 4 and 5A) of the DD, as well as in the activation loop and helix αG of the KD (Figures 3 and 5B). Within the DD, helices 2, 3, and 4 majorly interact with the other DDs of the myddosome complex. The KD helix αG movement may be important for forming appropriate interactions during the phosphorylation transition state catalyzed by the KD.

However, in the analysis of selective pressure for the IRAK members, site-specific positive selection amino acids were not identified in the DD or KD but were distributed in the ProST domain between the DD and KD, suggesting that the IRAK proteins had been subjected to strong purifying selection to become functionally distinct. In addition to these evolutionary and functional analyses, we investigated the structural similarities between the IRAKs by building 3D models of the DDs and KDs. These 3D models, particularly the DD models, were then subjected to MD simulations. Superimposition of the 3D models of the DDs and KDs showed that the IRAK paralogs are structurally similar. In addition, based on the structural analysis, we demonstrated that the KDs of IRAK4, TLK, and Pelle are structurally similar (Figure 7D) However, IRAK4 and TLK are RD-containing kinases. Moreover, the sequence similarity between the IRAK4 KD and TLK KD is higher than that between the Pelle KD and IRAK4 KD (Table S6), which clearly suggests that IRAK4 is a potential homolog of Tube/TLK rather than Pelle. In summary, our study provided useful information about the phylogeny and functional divergence of the IRAK gene family. The Tube/TLK, Pelle, PIK-1, IRAK1, IRAK2, and IRAKM subfamilies likely evolved from an ancient IRAK4-like kinase through gene duplication in metazoans. Acceleration of the asymmetric evolutionary rate and purifying selection of the new gene set were then likely the main contributors to gene stability.

This study represents the first detailed evolutionary and functional analysis of the IRAKs. The phylogenetic analysis suggests that IRAK4-like kinase is the ancestral gene of all IRAKs. Furthermore, the phylogenetic analysis indicates that all IRAK family members have been duplicated and have diverged from an ancestral gene in the metazoan lineage. In addition, our study indicates that the Tube protein from *Drosophila* is a homolog of the vertebrate IRAK4 protein. On the basis of 3D protein modeling, we propose that the IRAK proteins share a similar fold, likely retaining a similar function. However, functional divergence was predicted from the statistical evaluation of the IRAK family members, and the structural features were exploited. In conclusion, our study provides valuable insight into the IRAK gene family evolution that can be addressed experimentally at the molecular level in future studies.

## Supporting Information

Figure S1 **Phylogenetic relationship between vertebrate IRAK subfamilies determined using the NJ method.** A total of 139 IRAK sequences were included in this analysis. *A. queenslandica* was considered as an outgroup. The numerical values represent bootstrap values. As depicted, the tree is composed of 4 major monophyletic clades, and each clade is represented by a unique color. Colors are described in Figures 1 and 2. The species closest to the outgroup are invertebrate sequences and are shown in red. Taxonomic convention: the IRAK protein name followed by an abbreviated form of the species name.
(TIF)

Figure S2 **Phylogenetic analysis of IRAK genes with the corresponding** ***D. melanogaster*** **homologs determined using the NJ method.** A total of 73 sequences were included in this analysis (22 representative vertebrate IRAK subfamily sequences by taxonomy; 19 Pelle, 26 Tube/TLK, and 6 PIK-1 sequences). *A. queenslandica* was considered as an outgroup. The numerical values represent bootstrap values. Each IRAK subfamily clade is represented by a unique color. Colors are described in Figures 1 and 2. Taxonomic convention: the protein name followed by an abbreviated form of the species name.
(TIF)

Figure S3 **Death domain comparative models.** Death domain (DD) structures are shown. Type I potential sites for IRAK1, IRAK2, and IRAKM are shown using a ball-and-stick representation. Most of the Type I sites are located in helix 3 and helix 4.
(TIF)

Figure S4 **RMSD of the IRAK DD backbone atoms during MD simulations.** RMSD of Cα-back bone atoms with respect to the initial structure shows the stable nature of the DDs after the equilibration time.
(TIF)

Figure S5 **Comparative modeling of kinase domains.** Six KD models were constructed using comparative modeling (IRAK1, IRAK2, IRAKM, Pelle, TLK, and PIK-1). Type I potential sites for IRAK1, IRAK2, IRAKM, and IRAK4 are shown in a ball-and-stick representation. The sites are evenly distributed in conserved structural elements.
(TIF)

Table S1 **IRAK** homologs used for phylogenetic analysis. The table lists the molecular features of all IRAK homologs (n = 193, vertebrate and insect sequences) identified in public databases that were utilized for IRAK phylogenetic tree reconstructions. Species names followed by “*” are not included in the tree construction as it shows poor alignment. NP_BIND (extent of nucleotide phosphate-binding region); BINDING (binding site for any chemical group); ACT_SITE (amino acid involved in the enzyme).
(XLSX)

Table S2 **Inter- and intra-group sequence similarity among IRAK family members.** All-against-all pairwise similarity distances between the IRAK sequences were determined using a MAFFT alignment. The numbers included in parentheses below the names of the IRAK family members indicate the number of sequences included in the analysis of each subfamily.
(DOC)

Table S3 **Comparative analysis.** The table lists TRAF6, TRAF2, ubiquitination, and PEST motifs for all vertebrate IRAK members included in the phylogenetic analysis.
(XLSX)

Table S4 **Type I potential sites.** The table lists all type I potential sites for the death domains (DDs) and kinase domains (KDs) of each cluster of IRAKs.
(DOCX)

Table S5 **Model evaluation.** The table illustrates the model evaluation scores for all the models before and after energy minimization. The displayed IRAK models are reliable in terms of the overall packing. ProQ_LG: >1.5 fair; >2.5 good; >4 excellent. ProQ_MX: >0.l fair; >0.5 good; >0.8 excellent. MetaMQAP_GDT/RMSD: an ideal model has a GDT score >59 and an RMSD of approximately 2.0 Å.
(DOCX)

Table S6 **Intra- and inter-species kinase domain (KD) similarity.** All-against-all pairwise similarity distances between the KDs of the IRAK sequence based on a MAFFT alignment. Below the table, the inter-species similarities between TLK and IRAK4 are shown.
(DOCX)
