# Supplementary figures and images for: Molecular Evolution and Structural Features of IRAK Family Members
Source: PLoS One. 2012 Nov 14;7(11):e49771. doi: 10.1371/journal.pone.0049771 (PMC3498205; doi:10.1371/journal.pone.0049771)

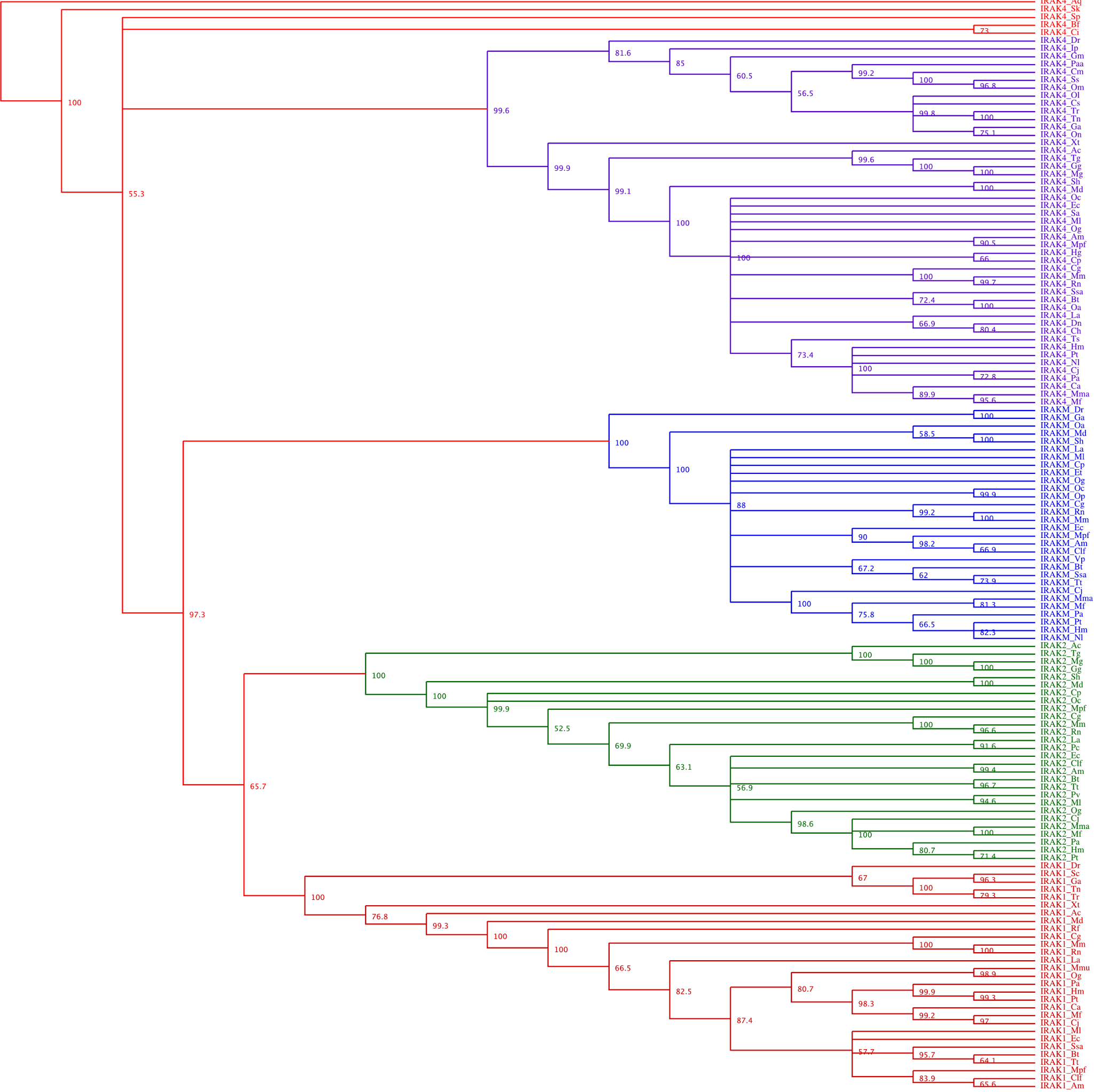

Supplement: Figure S1 — Phylogenetic relationship between vertebrate IRAK subfamilies determined using the NJ method. A total of 139 IRAK sequences were included in this analysis. A. queenslandica was considered as an outgroup. The numerical values represent bootstrap values. As depicted, the tree is composed of 4 major monophyletic clades, and each clade is represented by a unique color. Colors are described in Figures 1 and 2. The species closest to the outgroup are invertebrate sequences and are shown in red. Taxonomic convention: the IRAK protein name followed by an abbreviated form of the species name. (TIF) [file pone.0049771.s001.tif]

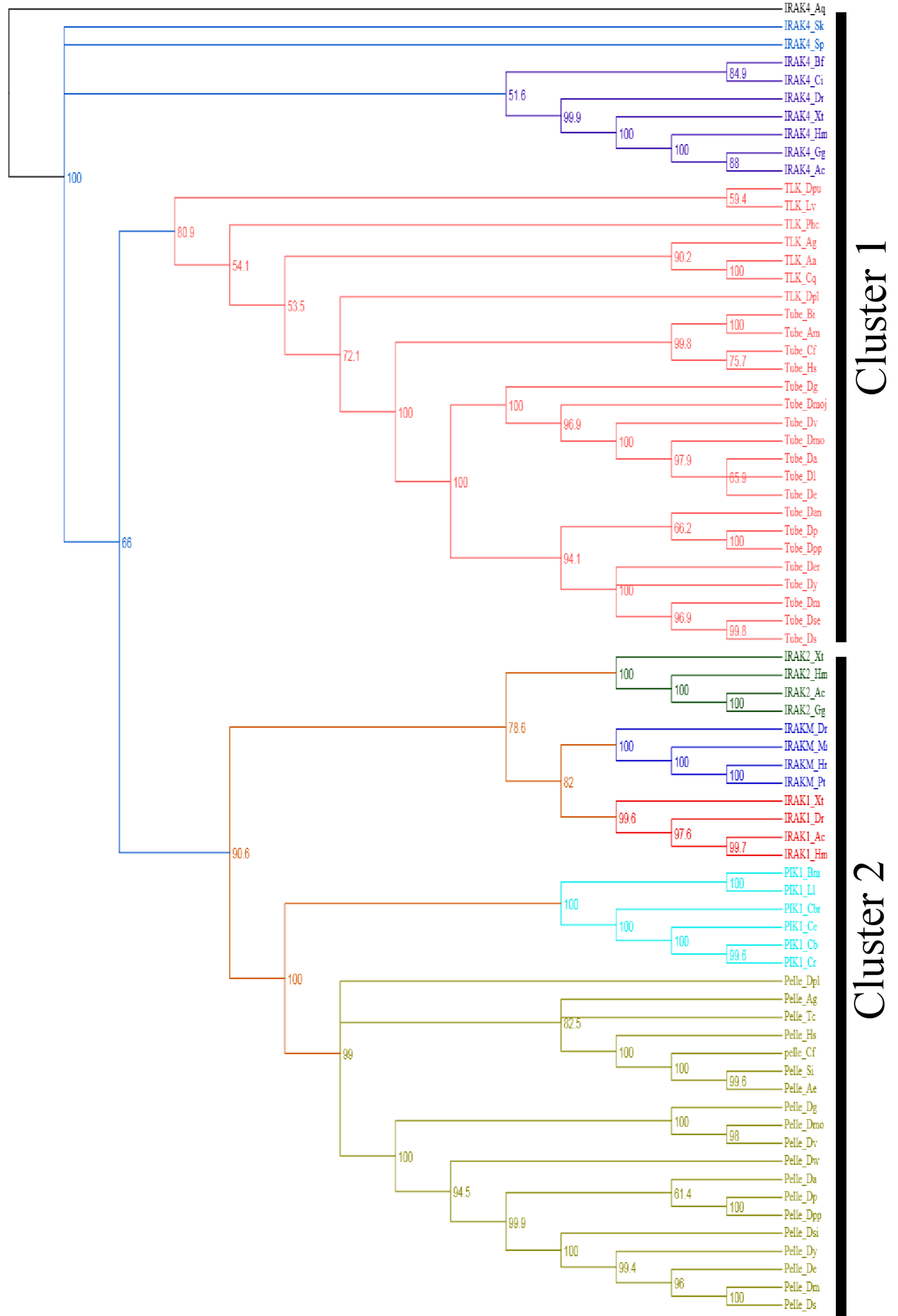

Supplement: Figure S2 — Phylogenetic analysis of IRAK genes with the corresponding D. melanogaster homologs determined using the NJ method. A total of 73 sequences were included in this analysis (22 representative vertebrate IRAK subfamily sequences by taxonomy; 19 Pelle, 26 Tube/TLK, and 6 PIK-1 sequences). A. queenslandica was considered as an outgroup. The numerical values represent bootstrap values. Each IRAK subfamily clade is represented by a unique color. Colors are described in Figures 1 and 2. Taxonomic convention: the protein name followed by an abbreviated form of the species name. (TIF) [file pone.0049771.s002.tif]

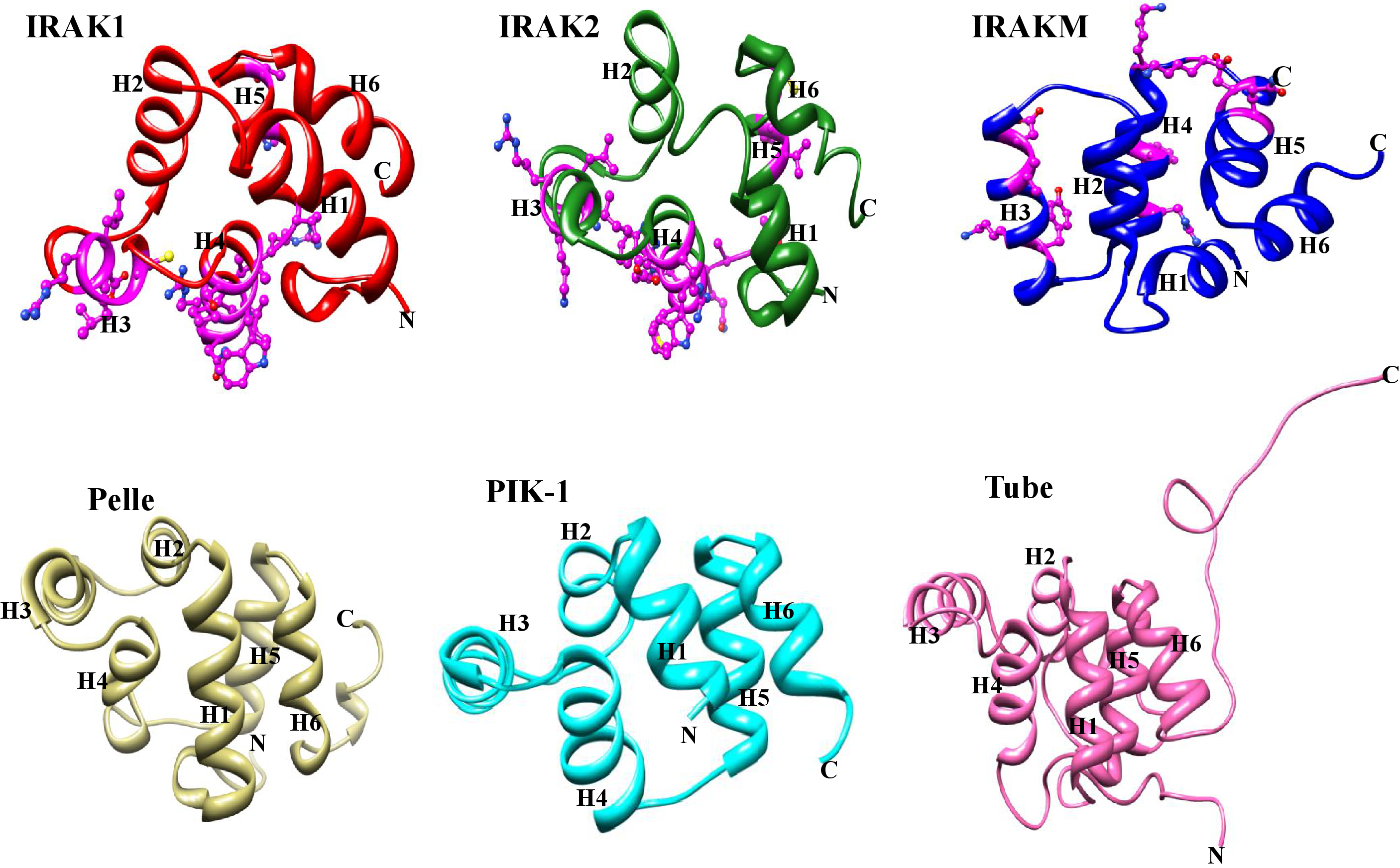

Supplement: Figure S3 — Death domain comparative models. Death domain (DD) structures are shown. Type I potential sites for IRAK1, IRAK2, and IRAKM are shown using a ball-and-stick representation. Most of the Type I sites are located in helix 3 and helix 4. (TIF) [file pone.0049771.s003.tif]

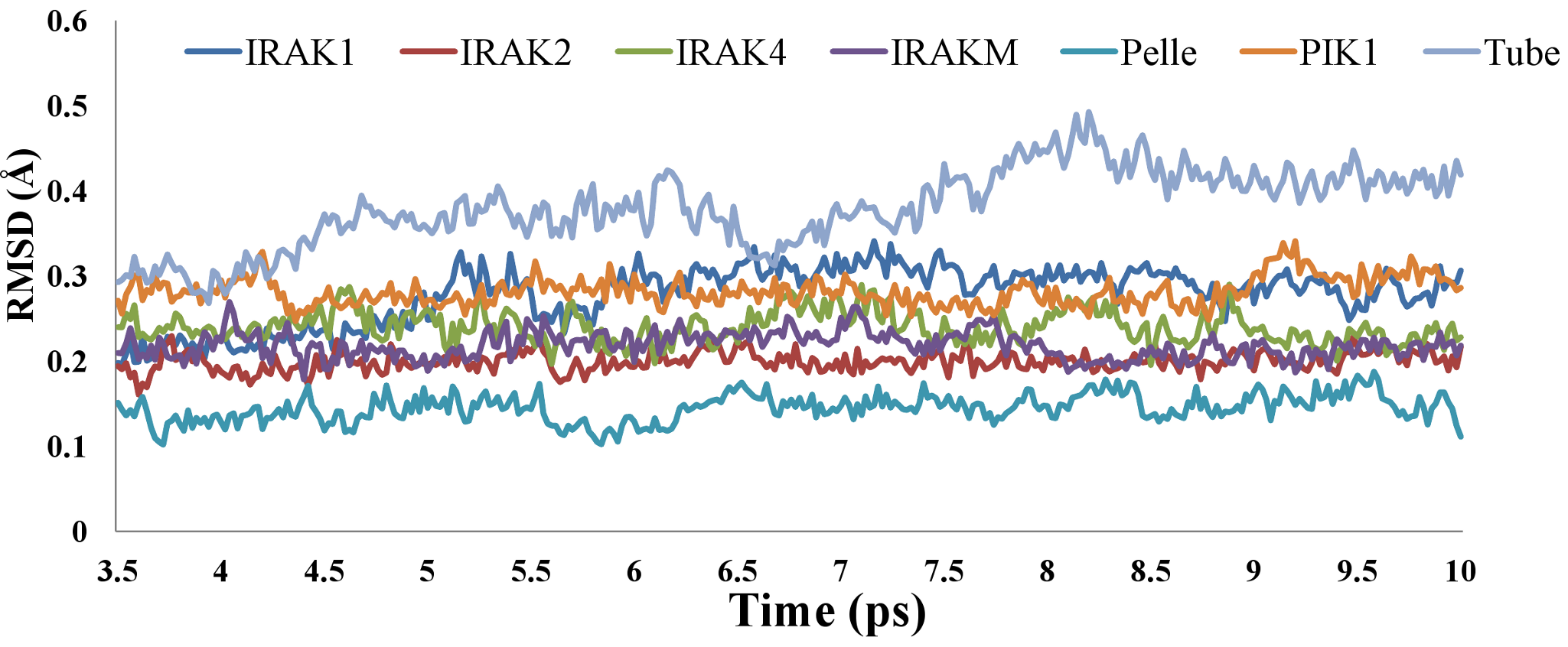

Supplement: Figure S4 — RMSD of the IRAK DD backbone atoms during MD simulations. RMSD of Cα-back bone atoms with respect to the initial structure shows the stable nature of the DDs after the equilibration time. (TIF) [file pone.0049771.s004.tif]

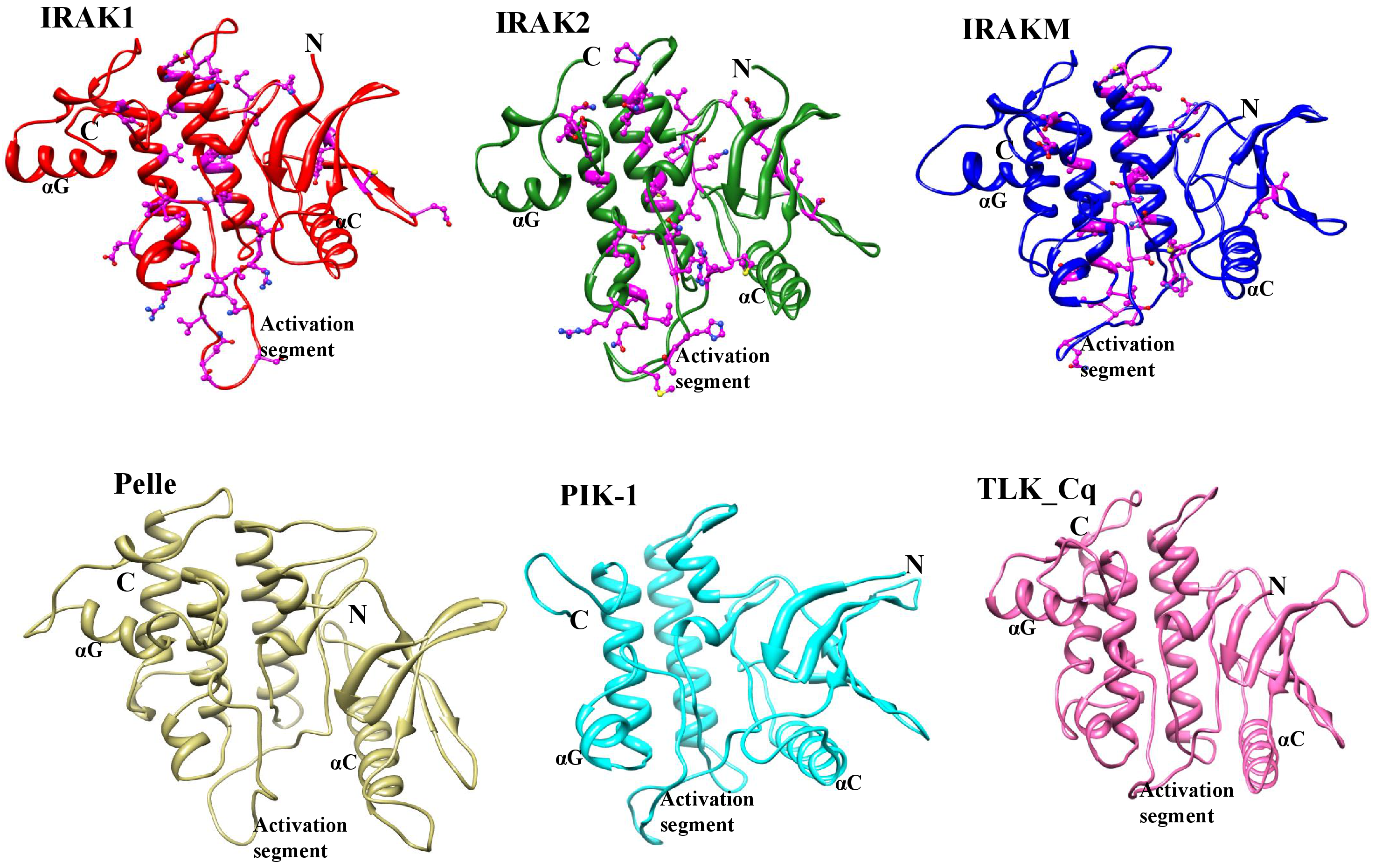

Supplement: Figure S5 — Comparative modeling of kinase domains. Six KD models were constructed using comparative modeling (IRAK1, IRAK2, IRAKM, Pelle, TLK, and PIK-1). Type I potential sites for IRAK1, IRAK2, IRAKM, and IRAK4 are shown in a ball-and-stick representation. The sites are evenly distributed in conserved structural elements. (TIF) [file pone.0049771.s005.tif]
