# Supplementary material for: Molecular Evolution and Structural Features of IRAK Family Members
Source: PLoS One. 2012 Nov 14;7(11):e49771. doi: 10.1371/journal.pone.0049771 (PMC3498205; doi:10.1371/journal.pone.0049771)
Supplement: Table S2 — Inter- and intra-group sequence similarity among IRAK family members. All-against-all pairwise similarity distances between the IRAK sequences were determined using a MAFFT alignment. The numbers included in parentheses below the names of the IRAK family members indicate the number of sequences included in the analysis of each subfamily. (DOC) [file pone.0049771.s007.doc]

|  | **IRAK1** | **IRAK2** | **IRAKM** | **IRAK4** | **Pelle** | **Tube** | **TLK** | **PIK-1** |
| --- | --- | --- | --- | --- | --- | --- | --- | --- |
| **IRAK1**  **(29)** | 30.4-99.1 | 21.3-26.2 | 18.5-24.1 | 17.6-24.2 | 16-22.7 | 4-7.8 | 13.1-21.2 | 16.2-21 |
| **IRAK2**  **(28)** | 19.4-25.3 | 40.5-97.4 | 15.1-19.8 | 15.3-25.8 | 13.9-19.2 | 4.3-6.6 | 11.9-19.2 | 14.7-17.8 |
| **IRAKM**  **(30)** | 17.3-24.5 | 17-24.4 | 38.4-99.9 | 17.9-27.9 | 16.6-22.2 | 5.9-8.6 | 12.5-21.5 | 16.9-21.9 |
| **IRAK4**  **(52)** | 21.3-26.9 | 19.5-24.3 | 23.7-29.5 | 29-99.8 | 21-26.4 | 6.1-9.7 | 16.6-30.6 | 23.6-25 |
| **Pelle**  **(19)** | 17.8-23.3 | 15.3-19.9 | 18.1-27.1 | 15.7-23.3 | 29.4-99.5 | 27.7-30.2 | 12.7-23.4 | 6-9.1 |
| **Tube**  **(19)** | 5.2-9 | 3.8-6.3 | 5.6-9 | 5.7-9.1 | 6.6-8.8 | 12.7-97 | 7.2-12.4 | 6.7-8.2 |
| **TLK**  **(7)** | 13.4-18.3 | 11.8-18.4 | 12.8-15.6 | 12.7-17.9 | 13.9-16.3 | 7.2-10.3 | 16.3-42.2 | 14.1-15.4 |
| **PIK-1**  **(6)** | 18.4-25 | 14.9-19.5 | 16.8-26.2 | 16.4-27 | 26.1-31.9 | 4.6-7.6 | 13.3-23 | 36-78.6 |
