# Supplementary material for: Molecular Evolution and Structural Features of IRAK Family Members
Source: PLoS One. 2012 Nov 14;7(11):e49771. doi: 10.1371/journal.pone.0049771 (PMC3498205; doi:10.1371/journal.pone.0049771)
Supplement: Table S4 — Type I potential sites. The table lists all type I potential sites for the death domains (DDs) and kinase domains (KDs) of each cluster of IRAKs. (DOCX) [file pone.0049771.s009.docx]

IRAK4/IRAK1(0.8)

IRAK4🡪52H,53I,56F,69E,72F,73D,75G,76T,77T,78N,79C,80T,82G,85V,86D,89I

IRAK1🡪70E,71L,74C,83S,86W,87P,89I,90N,91R,92N,93A,94R,96A,98V,98H,101T

IRAK4/IRAK2 (0.8)

IRAK4🡪53I, 54R, 55R, 68C, 72F, 75G, 76T, 77T, 79C, 85V

IRAK2🡪 42L,43R, 44K,58R,62W,65G,66M,67R,69A,75V

IRAK4/IRAKM(0.5)

IRAK4🡪52H,53I,58A,59L,69C,73F,78T,80C,84D,87D,88L

IRAKM🡪54D,55V,60K,61Y,70R,74W,79K,81K,85D,88Q,89V

IRAK1/IRAK2(0.75)

Irak2🡪41Q,43R,44K,45I,58R,59E,62W,63W,68Q,69A,70T,75V,76D,79C

IraK1🡪68E,70R,71L,72C,81A,82S,85W,86P,91N,92A,93R,98V,99H,102T

**Type I potential sites for KDs of Vertebrate IRAK subfamily (cluster pairwise comparison)**

IRAK2/IRAKM (0.8)

IRAK2🡪272L,125Q,360P,309Q,313I,317L,334S,335N,348T,350K,356A,357H,372T,376R,394K,417N

IRAKM🡪228A,256V,263PS,266I,270IV,274I,292G,293S,306Q,308K,315A,316H,331T,335S,353I,376D

IRAK2/IRAK1(0.8)

IRAK2🡪283S,286Y,287P,299Q,306LP,313I,321CR,324E,334SG,355LM,356A,357H,369M,373H,379LA,382IL,391Q,393T,405L,410T,416D

IRAK1🡪285C,288Y,289G,303Q,311S,318I,324R,327Q,339G,361L,362A,363R,378V,382QR,388L,391L,400R,402A,414V,419A,425K

IRAK2/IRAK4(0.8)

IRAK2🡪287P,288Y,290AP,298G,303Q,334SG,347LF,349P,355M,356A,357H,369M,391Q,392L,417N

IRAK4🡪263V,264Y,266P,276C,277L,287M,310R,323F,325A,332L,333A,334R,343V,363E,364I,389E

IRAKM/IRAK1(0.9)

IRAKM🡪255C,266I,274I,279H,293S,306Q,321l,324Q,331T,335S,344E,354K,364I,369T

IRAK1🡪302C,314Q,322T,327Q,340D,353T,371S,374Q,381T,383T,393E,404D,414V,419A, IRAKM/IRAK4(0.7)

IRAKM->239L,265H,275S,286P,304Q,314M,331T,335S,337S,351L,357I

IRAK4->260L,286H,296A,304E,322A,332L,345T,347R,349V,363E,370I

IRAK1/IRAK4 (cutoff 0.8)

IRAK4->389E,382T,346S,336S,323F,300NS,287M,286H,276C,266P,254D

IRAK1->425T,419A,382Q,371S,352L,327Q,314Q,313P,302C,292P,280Q
