# Supplementary material for: Molecular Evolution and Structural Features of IRAK Family Members
Source: PLoS One. 2012 Nov 14;7(11):e49771. doi: 10.1371/journal.pone.0049771 (PMC3498205; doi:10.1371/journal.pone.0049771)
Supplement: Table S5 — Model evaluation. The table illustrates the model evaluation scores for all the models before and after energy minimization. The displayed IRAK models are reliable in terms of the overall packing. ProQ_LG: >1.5 fair; >2.5 good; >4 excellent. ProQ_MX: >0.l fair; >0.5 good; >0.8 excellent. MetaMQAP_GDT/RMSD: an ideal model has a GDT score >59 and an RMSD of approximately 2.0 Å. (DOCX) [file pone.0049771.s010.docx]

| Models (DD) | ProQ_LG/MX | | MetaMQAP_GDT/RMSD | |
| --- | --- | --- | --- | --- |
|  | Before Energy minimization | After Energy minimization | Before Energy minimization | After Energy minimization |
| IRAK1 | 3.901/0.609 | 4.001/0.609 | 65.546/2.214 | 66.389/2.068 |
| IRAKM | 2.632/0.465 | 2.836/0.473 | 58.715/2.521 | 61.709/2.497 |
| PIK-1 | 3.7128/0.444 | 3.736/0.477 | 56.142/3.045 | 60.987/2.887 |
| Models(KD) |  |  |  |  |
| IRAK1 | 2.226/0.241 | 2.454/0.256 | 49.365/3.812 | 51.885/3.70 |
| IRAK2 | 3.215/0.301 | 3.217/0.304 | 52.614/3.90 | 54.828/3.738 |
| IRAKM | 4.156/0.4152 | 4.247/0.442 | 66.201/2.510 | 70.055/2.499 |
| Pelle | 2.631/0.251 | 2.952/0.272 | 45.146/4.01 | 49.640/3.812 |
| TLK_Cq | 2.312/0.202 | 2.403/0.212 | 54.320/3.645 | 56.827/3.436 |
| PIK-1 | 2.513/0.316 | 2.709/0.317 | 49.315/2.916 | 52.885/2.805 |

The displayed IRAK models are reliable in terms of the overall packing. ProQ_LG: >1.5 fair; >2.5 good; >4 excellent. ProQ_MX: >0.l fair; >0.5 good; >0.8 excellent. MetaMQAP_GDT/RMSD: an ideal model has a GDT score >59 and an RMSD of approximately 2.0 Å.
