# Supplementary material for: Molecular Evolution and Structural Features of IRAK Family Members
Source: PLoS One. 2012 Nov 14;7(11):e49771. doi: 10.1371/journal.pone.0049771 (PMC3498205; doi:10.1371/journal.pone.0049771)
Supplement: Table S6 — Intra- and inter-species kinase domain (KD) similarity. All-against-all pairwise similarity distances between the KDs of the IRAK sequence based on a MAFFT alignment. Below the table, the inter-species similarities between TLK and IRAK4 are shown. (DOCX) [file pone.0049771.s011.docx]

|  | **IRAK-1** | **IRAK-2** | **IRAK-M** | **IRAK-4** | **Pelle** | **TLK** | **PIK-1** |
| --- | --- | --- | --- | --- | --- | --- | --- |
| **IRAK-1** | 41.5-99.1 | 29.3-36.2 | 32.1-42.4 | 30.1-41.1 | 28.8-36.4 | 23.5-35.5 | 29.6-34.7 |
| **IRAK-2** | 27.1-29.9 | 51.7-99.1 | 22.5-31.3 | 26.9-31.5 | 21.5-30.2 | 18.1-26.9 | 23.4-28.3 |
| **IRAK-M** | 31.2-38.8 | 26.9-33.2 | 46.5-98.5 | 26-31.3 | 26.5-35.4 | 23.7-30.9 | 26.6-32.8 |
| **IRAK-4** | 31.5-35.5 | 27-34.9 | 26.1-36.2 | 52.7-99.6 | 32.3-44.8 | 31.1-44.4 | 32.4-42.1 |
| **Pelle** | 28.5-32 | 21-28.8 | 25.6-34.2 | 29.3-38.8 | 36-99.5 | 25.3-36.3 | 36-44.7 |
| **TLK-Ag** | 28.7-32 | 18.2-27.2 | 23.9-30.6 | 32.9-39.2 | 26.7-31.5 | 26.8-55.9 | 23.5-29.4 |
| **PIK-1** | 34.3-38.3 | 23.9-31.1 | 24-37.2 | 36.4-41.5 | 41.2-52.7 | 25.2-39.3 | 51.1-87.4 |

TLK-Cq—28.4 to 37.1, TLK-Aa 28.1-33.6, TLK- Dp 28.1-34.1, TLK-Phc-33.5-44.4, TLK- Lv-41.2-52.8, TLK-Dpu- 36.7-42
